# How Many Patients with Type 2 Diabetes Meet the Inclusion Criteria of the Cardiovascular Outcome Trials with SGLT2 Inhibitors? Estimations from a Population Database in a Mediterranean Area

**DOI:** 10.1155/2019/2018374

**Published:** 2019-11-11

**Authors:** Silvia Canivell, Manel Mata-Cases, Bogdan Vlacho, Mònica Gratacòs, Jordi Real, Dídac Mauricio, Josep Franch-Nadal

**Affiliations:** ^1^DAP-Cat Group, Unitat de Suport a la Recerca Barcelona, Fundació Institut Universitari per a la recerca a l'Atenció Primària de Salut Jordi Gol i Gurina (IDIAPJGol), Barcelona, Spain; ^2^Primary Health Care Center Sant Martí de Provençals, Gerència d'Atenció Primaria, Institut Català de la Salut, Barcelona, Spain; ^3^Health Sciences Research Institute and Hospital Universitari Germans Trias i Pujol, Badalona, Spain; ^4^CIBER of Diabetes and Associated Metabolic Diseases (CIBERDEM), Instituto de Salud Carlos III (ISCIII), Barcelona, Spain; ^5^Primary Health Care Center La Mina, Gerència d'Àmbit d'Atenció Primària Barcelona Ciutat, Institut Català de la Salut, Sant Adrià de Besòs, Spain; ^6^Department of Endocrinology & Nutrition, Hospital de la Santa Creu i Sant Pau, Autonomous University of Barcelona, Barcelona, Spain; ^7^Primary Health Care Center Raval Sud, Gerència d'Atenció Primaria, Institut Català de la Salut, Barcelona, Spain

## Abstract

**Objective:**

Regulatory agencies require the assessment of cardiovascular (CV) safety for new type 2 diabetes (T2D) therapies through CV outcome trials (CVOTs). However, patients included in CVOTs assessing sodium-glucose cotransporter-2 inhibitors (SGLT2i) might not be representative of those seen in clinical practice. This study examined the proportion of patients that would have been enrolled into three main SGLT2i CVOTs to determine whether these trials' eligibility criteria can be applied to a real-world Mediterranean T2D population.

**Methods:**

Cross-sectional, retrospective, cohort study of T2D patients registered in primary care centres of the Catalan Institute of Health using medical records from a population database (SIDIAP) that includes approximately 74% of the population in Catalonia (Spain). Eligibility criteria were according to those of three SGLT2i CVOTs: EMPA-REG OUTCOME (empagliflozin), CANVAS (canagliflozin), and DECLARE-TIMI 58 (dapagliflozin).

**Results:**

By the end of 2016, the database included 373,185 patients with T2D with a mean age of 70 ± 12 years, 54.9% male, with a mean duration of T2D of 9 ± 6 years, and a mean glycated haemoglobin (HbA1c) of 7.12% ± 1.32 (59% with HbA1c < 7%). Of these, 86,534 (23%) had established CV disease and 28% chronic renal failure (estimated glomerular filtration < 60 ml/min/1.73m^2^). Among all included patients, only 8.2% would have qualified for enrolment into the EMPA-REG OUTCOME trial, 29.6% into the CANVAS program, and 38% into the DECLARE-TIMI 58 trial. The main limiting factors for inclusion would have been a previous history of CV disease and the baseline HbA1c value.

**Conclusion:**

The external validity of the analysed CVOTs is clearly limited when applying the same eligibility criteria to a T2D Mediterranean population.

## 1. Introduction

Patients with type 2 diabetes (T2D) have an increased risk of renal and cardiovascular disease (CVD) and mortality [1]. Therefore, improvement in cardiovascular (CV) health is one of the main goals of diabetes management. While tight good glycaemic control in T2D is associated with reduced risk of microvascular disease [2, 3], the benefit regarding macrovascular disease is less clear [4–6]. Indeed, a meta-analysis combining the results of large-scale trials showed that intensive glucose-lowering therapy was associated with a significant reduction in the overall incidence of CV events and myocardial infarction compared to conventional therapy (odds ratio (OR) 0.89, *P* = 0.001; OR 0.84, *P* < 0.001, respectively) [7]. However, there was no difference in the incidence of CV mortality [7]. Both the US Food and Drug Administration (FDA) and the European Medicines Agency (EMA) require, for each new antidiabetic therapy to treat T2D, to show a neutral or beneficial effect in CV safety through the conduction of CV outcome trials (CVOTs) [8, 9].

Sodium-glucose cotransporter-2 inhibitors (SGLT2i) are a promising group of new drugs for the treatment of T2D that act by preventing the reabsorption of glucose from the proximal renal tubule in the kidney [10]. Additionally, they have numerous pleiotropic effects such as reducing blood plasma glucose, body weight, and blood pressure and inducing natriuresis [10]. In the particular case of SGLT2i, recent CVOTs have shown renal and CV benefits and further studies are ongoing [11–13]. However, one of the major issues of randomised clinical trials (RCTs) is the external validity of the results, that is, to what extent the overall average effect of the treatment can be generalised to a particular group of patients or clinical setting [14]. For instance, the external validity can be challenged by the trial's setting (e.g., differences between countries regarding the health care system, disease management, or natural history of the disease), the inclusion and exclusion criteria, or differences between the protocol trial and routine clinical practice, among other issues [14].

The results of the CVOTs of three SLGT2 inhibitors available in Spain published to date are EMPA-REG OUTCOME with empagliflozin [15], CANVAS with canagliflozin [16], and DECLARE-TIMI 58 with dapagliflozin [17]. The EMPA-REG OUTCOME trial included only patients with established CV disease (CVD), i.e., secondary prevention [15]. The other two trials included secondary prevention patients and also patients with CV risk factors who have not yet developed CVD (primary prevention): with ≥1 CV risk factors in the DECLARE-TIMI 58 trial [17] and with ≥2 CV risk factors in the CANVAS study [16]. Since the eligibility criteria varied among these SGLT2i CVOTs, it was expected that the external validity of the different studies might also differ; thus, the trial population does not actually represent the general T2D population. Indeed, the external validity of CVOTs regarding SGLT2i has been assessed by two recently published studies using clinical routine data from the US and Northern Europe [18, 19]. Both studies found large differences between trials regarding the proportion of patients seen in clinical practice that would have met entry criteria in these CVOTs, with the DECLARE-TIMI 58 trial as the most generalisable and applicable one. Moreover, the results from the study conducted in Northern Europe were consistent across all four included countries (i.e., Germany, The Netherlands, Norway, and Sweden) [18]. However, there is no published information from Southern European countries so far, although the distribution of CV risk factors as well as the prevalence of CVD in patients with diabetes differs across regions in Europe [20, 21]. Based on these potential differences, we hypothesised that the external validity of the CVOTs could be different when the general T2D population is estimated in a Mediterranean country.

The aim of the present study was to determine the proportion of patients with T2D in primary care that would be eligible for inclusion in the CVOTs of SLGT2i in the population served by the Catalonian Health Institute in Catalonia, a Mediterranean area in the northeast of Spain.

## 2. Materials and Methods

### 2.1. Design

This was a cross-sectional retrospective study of the T2D population attended at primary care centres of Catalonia, an autonomous region located in the northeast of Spain, corresponding to 12% of the total Spanish population. We compared the potential eligibility of patients to those included in publications describing three completed SLGT2i CVOTs [15–17].

The study was approved by the Ethics Committee of the Primary Health Care University Research Institute (IDIAP) Jordi Gol in accordance with the Spanish regulations on observational studies. This retrospective study using anonymised data did not require obtaining informed consent from the patients.

### 2.2. Data Source

Data from patients were extracted from the SIDIAP database, which contains anonymised longitudinal patient information obtained from electronic clinical records; it incorporates available information from 288 primary care teams of the Catalonian Health Institute (ICS), which serves around 5.6 million people, 74% of the total population in Catalonia. The SIDIAP includes demographic, clinical, and pharmacy-invoicing data provided by the CatSalut general database, and it has already been used for epidemiological research purposes and real-world evidence [22–24].

### 2.3. Inclusion and Exclusion Criteria

The study population consisted of patients aged 18 years or older with a diagnosis of T2D (International Classification of Diseases (ICD-10) codes E11, E11.0-E11.9, E14, and E14.0-E14.9) as of 31 December 2016 (index date). We excluded patients with a diagnosis of type 1 diabetes, gestational diabetes mellitus, and any other type of diabetes. After this initial selection, we applied the inclusion and exclusion criteria from the CVOTs for the SGLT2i commercialised up to date in Spain, namely, empagliflozin (EMPA-REG OUTCOME trial), canagliflozin (CANVAS trial), and dapagliflozin (DECLARE-TIMI 58 trial) (Supplementary [Supplementary-material supplementary-material-1]) [15–17].

### 2.4. Study Variables

The following variables from the SIDIAP database at the end of December 2016 were analysed: age and gender; duration of T2D; the most recent value (closest to 31 December 2016) of glycated haemoglobin (HbA1c); and presence of risk factors, including hypertension (ICD-10 codes I10 and I15, or systolic blood pressure (SBP) ≥ 140 and/or diastolic blood pressure (DBP) ≥ 90 mmHg, or use of antihypertensive medications), dyslipidemia (ICD-10 codes E780 to E785, or LDL cholesterol (LDLc) ≥ 160 mg/dl, or use of lipid-lowering drugs), smoking status, body mass index (BMI), estimated glomerular filtration rate (eGFR) using the Chronic Kidney Disease Epidemiology Collaboration (CKD-EPI) equation, albumin-to-creatinine ratio (UACR), and history of CVD (stroke, peripheral artery disease, ischaemic heart disease, and heart failure) (Supplementary [Supplementary-material supplementary-material-1]). Noninsulin antidiabetic drugs (NIADs) and insulin-active electronic prescriptions on the index date were also considered.

### 2.5. Statistical Analysis

The eligibility was determined by dividing the number of patients fulfilling each of the CVOTs key inclusion and exclusion criteria by the total T2D registered population. Data were summarised as mean (standard deviation (SD)) or *n* (%).

## 3. Results

A total of 373,185 patients with T2D were registered in the SIDIAP database as of 31 December 2016 (Table 1). The mean age was 70.1 ± 12.3 years, and 54.9% were male. The mean T2D duration was 9.3 ± 6.2 years, and the mean HbA1c was 7.12% ± 1.32. More than half (59%) of the patients had good glycaemic control (HbA1c ≤ 7%), about one-third (28%) had chronic kidney disease (eGFR < 60 ml/min/1.73m^2^), and 4% had severe renal insufficiency (eGFR < 30 ml/min/1.73m^2^). Overall, 77% of patients (*n* = 286,651) did not have any ICD-10 code of established CVD recorded, thus considered primary prevention cases; 23% (*n* = 86,534) had an ICD-10 code of an established CVD, so they were considered secondary prevention cases. Patients with established CVD were more often men, older, and with a longer T2D duration and had more CV risk factors (Table 1).

**Figure 1:**
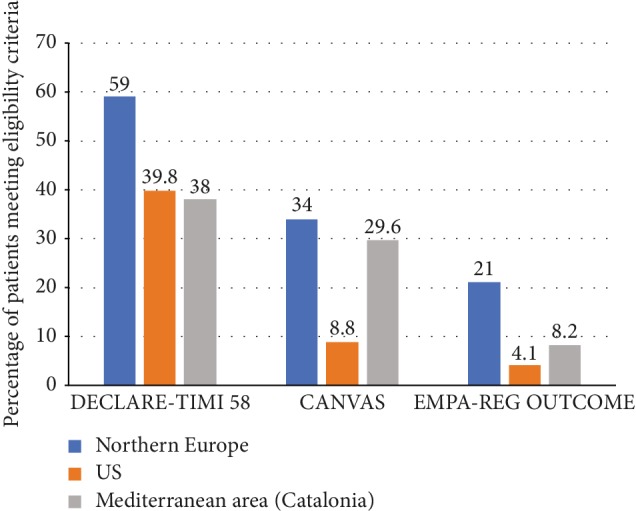
Graphical representation of the representativeness of patients in SGLT2i CVOTs when compared to the general type 2 diabetes population from four European countries, the US, and the present Mediterranean population.

Applying the EMPA-REG OUTCOME eligibility criteria, only 8.2% (*n* = 30,559) of the patients included in the SIDIAP database would have qualified for entry in the trial (Table 2), while 29.6% (*n* = 110,551) could have been enrolled in the CANVAS program (Table 3) and 38% (*n* = 141,653) would have been eligible for the DECLARE-TIMI 58 trial (Table 4). In Figure 1, these results are shown in comparison to those reported by two studies conducted in the US and Northern Europe [18, 19].

Compared with the characteristics of the patients enrolled in the three studied CVOTs (Table 5), the main limiting factors for inclusion would have been the absence of a history of CVD and the value of HbA1c. Specifically, only 23% of patients in our T2D population had a preexisting CVD and only 41% had HbA1c values ≥ 7%. Finally, chronic renal failure (present in 28% of our T2D patients) was a restrictive criterion in DECLARE, while in EMPAREG and CANVAS, only patients with severe renal failure (eGFR < 30 ml/min/1.73m^2^) were excluded (4% in SIDIAP).

## 4. Discussion

In this population-based study using routine clinical data, we estimated the proportion of patients with T2D that would have been eligible for inclusion in the main CVOTs regarding the use of SGLT2i in our environment (Catalonia, Spain). We found that the DECLARE-TIMI 58 CVOT trial had the highest representativeness, covering 38% of the T2D patients in our general T2D population, which is in line with previously reported studies conducted in Northern Europe and the US [13, 17, 25]. This result is not surprising since the DECLARE-TIMI 58 CVOT trial included similar proportions of patients in primary CV prevention and secondary CV prevention (59% and 41%, respectively) [17], which ensures the highest representativeness. The CVOT with the lowest generalisability was the EMPA-REG OUTCOME trial because it included only secondary prevention patients [15]. However, if we only take into account patients with cardiovascular disease as eligible to enter into the trials, the resulting proportion would have been similar among the three trials: 8.2% for the EMPAREG, 8.5% for the CANVAS program, and 7.9% for the DECLARE trial. Indeed, the main differences in the design of the different CVOT trials that we included were the enrolment of patients at high CV risk at baseline: EMPA-REG OUTCOME included only patients with T2D and established CV disease (i.e., secondary prevention), while both the CANVAS and the DECLARE-TIMI 58 also included primary prevention patients. In our real-world T2D population, the main limiting factors for inclusion in the different CVOTs analysed were the absence of a history of CV disease (present in the 23% of the patients) and glycaemic control: only 41% of the patients had a HbA1c ≥ 7% (entry criteria for the EMPA-REG OUTCOME and CANVAS trials), and 65.3% were above 6.5% threshold (entry criterion in the DECLARE-TIMI 58 study).

Concern regarding poor external validity (i.e., generalisability) of RCTs is largely known and has implications for the use (or underuse) of treatments in routine clinical practice [14, 26]. This topic has also been addressed in the past regarding RCTs in the field of diabetes. For instance, in a previous literature review, the external validity of large trials assessing the impact of glycaemic control on CVD in patients with T2D was reported as limited when applied to a T2D population-based cohort [27]. Another recent study evaluated the population representativeness of 1691 registered T2D trials [28] and found that in 51.4% of cases (and 53.1% of phase 2 and 3 interventional trials), the population representativeness was <5%. Of note, and in line with our results, the eligibility criterion that had the largest effect on the population representativeness was HbA1c [28]. Finally, the study showed that the greater the number of eligibility criteria, the lower the representativeness was, and the authors concluded that the low representativeness of T2D trials could be attributed to safety concerns when designing the study, which may lead to overly restrictive eligibility criteria to prevent adverse events [28].

Chronic renal failure was a restrictive criterion in DECLARE, while EMPA-REG OUTCOME and CANVAS trials only excluded patients with severe renal insufficiency (<30 ml/min/1.73 m^2^), present in 4% of our population. However, renal benefits of SGLT2i have been shown in different studies and one meta-analysis [29], particularly in the EMPA-REG OUTCOME and CANVAS trials [15, 16, 30–32], although these results were from secondary outcomes. New trials are ongoing with the primary renal endpoint (kidney outcome trials) focusing on patients with T2D and established chronic kidney disease (CKD) [33, 34]. The latest recommendations from the 2018 ADA/EASD Consensus Guidelines suggest to consider the use of a SGLT2i in patients with T2D and CKD [35]. In fact, the FDA has recently modified this limitation for patients with moderate renal impairment (i.e., eGFR45 60 ml/min/1.73 m^2^) for dapagliflozin, as previously done with canagliflozin 100 mg and empagliflozin 10 mg [36]. In summary, in the US, SGLT2i are still not recommended when eGFR is less than 45 ml/min/1.73m^2^ and remain contraindicated in patients with severe renal impairment (eGFR < 30 ml/min/1.73 m^2^), end-stage renal disease, or on dialysis [36]. Conversely, the EMA has not yet modified the general restriction of eGFR < 60 ml/min/1.73 m^2^. However, it is likely that recommendations by drug agencies for SGLT2i in CKD will change in the future after the publication of the results from the ongoing kidney outcome trials (CREDENCE, DAPA-CKD, and EMPAGLIFLOZIN RENAL) [37]. In addition, recent results of the CREDENCE trial showed that the risk of kidney failure and renal or CV mortality was 30% lower in the group of patients receiving canagliflozin compared to placebo (HR for the primary composite outcome of end-stage kidney disease, doubling of serum creatinine, or renal or CV death = 0.70; 95% CI, 0.59–0.82) [38]. All the patients included in this trial had an eGFR between 30 and 90 ml/min/1.73m^2^ and albuminuria, and in a subgroup analysis, the renal benefit of canagliflozin was higher among patients with eGFR between 45 and 60 ml/min/1.73m^2^ (HR 0.52; 95% CI, 0.38–0.72) [38].

Indeed, a recent systematic review and trial-level meta-analysis of SGLT2i CVOT trials concludes that SGLT2i reduce the risk of worsening eGFR in a broad spectrum of T2D patients [39]. Considering the facts mentioned above, if SGLT2i were allowed to be prescribed to patients with eGFR>30 ml/min/1.73 m^2^, the proportion of patients eligible for inclusion in the DECLARE TIMI 58 in the SIDIAP database would have increased.

The results of the present study show that the patterns of external validity of SGLT2i CVOTs were limited, in line with the studies conducted in Northern Europe or the US [18, 19]. However, the proportion of eligible patients would have been less in our population than in Northern Europe for all three trials but higher than that in the US for the CANVAS and EMPAREG studies as shown in Figure 1. This could be explained by differences in the prevalence of CVD between regions and countries. Indeed, the prevalence of CVD is higher in countries from the north of Europe than from the south of Europe [21], but it is also probable that differences in the prevalence of specific CV risk factors by country and region have impacted our results. The low eligibility for CANVAS and EMPA-REG OUTCOME in the US study is striking, although the percentage of patients having CVD was similar to ours (23.7 and 23%, respectively). One explanation for this discordance could be related to the different methodologies used in the US study, which is an estimation based on data from two NHANES surveys. In this study, data from 20,293 volunteer subjects, 2,395 of whom had T2D, were extrapolated to 23,941,512 persons having T2D in the US [19]. For instance, these patients were younger than in the other databases: 59 years old in the US, 68 years in the European study, and 70 years in our study. As a result, the prevalence of CV risk factors was lower in our database (13.6% in the US vs. 28%). Conversely, the prevalence of CVD in the European study (43.7% in Germany, 34.8% in The Netherlands, 31.4% in Sweden, and 25.1% in Norway) was higher in our database (23%) and produced higher percentages of inclusion in the CANVAS and EMPA-REG OUTCOME trials [18].

On the other hand, the applicability of SGLT2i is certainly much wider than the strict entry criteria of CVOTs, as shown in several of studies and subanalyses reporting the additional benefits of SGLT2i, particularly in chronic kidney disease and heart failure [40]. For instance, real-world evidence (RWE) studies have also confirmed reductions in mortality (43% to 49%) and hospitalization for heart failure (40% to 51%) in hundreds of thousands of patients [40]. So far, the main RWE studies published are CVD-Real, CVD-Real 2, EASEL, and EMPRISE; these studies compared SGLT2i with other antidiabetic drugs, especially against DPP-4 inhibitors (CVD-Real 2 and EMPRISE). However, our study aimed at determining how many patients from our Mediterranean database would be eligible to enter in each CVOT, but not the applicability of SGLT2i in the whole diabetic population. Thus, even though we found a low percentage of patients that would have been enrolled in each of the cardiovascular outcome trials, we should not dismiss the additional benefits of using SGLT2i in the whole diabetic population in terms of heart failure benefit. Hence, our results should be taken with caution and should not drive the decision to prescribe or not an SGLT2i in routine practice without considering all clinical aspects and patient preferences. Finally, we should point out that, at the time of deciding whether to prescribe or not an SGLT2i, clinicians should also refer to the latest available evidence and updated guidelines for T2DM management [35, 41]. In this line, the 2018 Consensus Report of the American Diabetes Association (ADA) and the European Association for the Study of Diabetes (EASD) state that, in the presence of cardiovascular disease, empagliflozin or canagliflozin should be recommended if HbA1c levels are above target, usually above 7% [35]. Moreover, the recently published 2019 ESC Guidelines in collaboration with the EASD recommend the use of SGLT2i in case of established cardiovascular disease or in subjects at high or very high CV risk, without establishing any specific threshold for HbA1c [41].

This study has some limitations. The main limitations derive from its retrospective observational nature, which are common to all similarly designed studies using real-world databases. For instance, 12% and 10% of patients had no available HbA1c or eGFR values during the evaluated year, respectively. On the other hand, the strength of the present study is that it involves real-world data from a Mediterranean region where the prevalence of CV risk factors and CVD in patients with T2D is expected to be different from that in Northern Europe or the US [21].

## 5. Conclusions

This study shows that there are considerable differences in the external validity of the different CVOTs of SGLT2i when applying the same eligibility criteria to the T2D population of Catalonia. The DECLARE-TIMI 58 CVOT was the most generalisable, while the EMPA-REG OUTCOME and CANVAS trials were much less representative of real-world T2D patients. However, the clinical applicability of SGLT2i in routine practice goes beyond the strict inclusion criteria of CVOTs and it is important to consider all patient-centered aspects before decision-making in T2D management.

## Figures and Tables

**Table 1:** Clinical characteristics of patients with T2D registered in the SIDIAP database.

Characteristics	All population (*N* = 373,185)	Secondary prevention patients^∗^ (*N* = 86,534)	Primary prevention patients^∗∗^ (*N* = 286,651)
Gender, male, *n* (%)	204,707 (54.9)	56,882 (65.7)	147,825 (51.6)
Age (years), mean (SD)	70.1 (12.3)	74.8 (10.5)	68.7 (12.5)
Current smokers, *n* (%)	52,744 (14.1)	11,058 (12.8)	41,686 (14.5)
BMI (kg/m^2^), *n* (%)			
≥30	144,592 (44.9)	30,366 (40.3)	114,226 (46.4)
>45	3,905 (1.2)	521 (0.7)	3,384 (1.4)
Duration of diabetes (years), mean (SD)	9.3 (6.2)	10.9 (6.7)	8.8 (6.0)
HbA1c (%)^†^, mean (SD)	7.12 (1.32)	7.16 (1.32)	7.10 (1.33)
HbA1c ≤ 7%, *n* (%)	194,751 (59.0)	43,664 (56.6)	151,087 (59.8)
Hypertension, *n* (%)	268,394 (71.9)	70,026 (80.9)	198,368 (69.2)
Dyslipidemia, *n* (%)	223,785 (60.0)	56,194 (64.9)	167,591 (58.5)
eGFR (ml/min/1.73m^2^)^†^, *n* (%)			
≥60	241,958 (72)	45,598 (58)	196,360 (76)
30-60	80,978 (24)	27,651 (35)	53,327 (21)
<30	13,262 (4)	5,909 (7)	7,353 (3)
UACR ≥ 30 mg/g, *n* (%)	51,429 (13.8)	17,126 (19.8)	34,303 (12.0)
T2D treatment, *n* (%)			
No antidiabetic medication	68,681 (18.4)	12,178 (14.1)	56,503 (19.7)
NIAD monotherapy	138,615 (37.1)	28,582 (33.0)	110,033 (38.4)
NIADs in combination	86,508 (23.2)	18,557 (21.4)	67,951 (23.7)
Insulin±NIAD	79,381 (21.3)	27,217 (31.5)	52,164 (18.2)

BMI: body mass index; eGFR: estimated glomerular filtration rate; NIAD: noninsulin antidiabetic drug; SD: standard deviation; T2D: type 2 diabetes; UACR: urine albumin-to-creatinine ratio; HbA1c: glycated haemoglobin. ^∗^Secondary prevention: patients with established cardiovascular disease (ICD-10 codes for coronary heart disease, cerebrovascular disease, or peripheral arteriopathy). ^∗∗^Primary prevention: patients without any ICD-10 code for cardiovascular disease. ^†^There were 12% of missing data in the registration of HbA1c and 10% in the registration of eGFR.

**Table 2:** Eligibility criteria for the EMPA-REG OUTCOME (empagliflozin) trial and number of patients in the SIDIAP database that would have met criteria for enrolment.

Eligibility criteria in EMPA-REG OUTCOME trial	Potentially eligible patients from the SIDIAP database (*N* = 373,185)
Inclusion criteria	*n* (%)
Age ≥ 18 years	373,185 (100)
Preexisting CV event: CHD, angina, MI, stroke, and PAD	86,534 (23.2)
HbA1c level 7.0%-≤10.0%	33,270 (8.9)
Main exclusion criteria	*n* (%)
eGFR < 30 ml/min/1.73m^2^	2,488 (0.7)
BMI > 45 kg/m^2^	223 (0.06)
Total eligible, *n* (%)	**30,559 (8.2)**

CHD: coronary heart disease; CV: cardiovascular; eGFR: estimated glomerular filtration rate; MI: myocardial infarction; PAD: peripheral artery disease; HbA1c: glycated haemoglobin; BMI: body mass index. All the percentages refer to the proportion from the total number of eligible subjects of the SIDIAP database (*n* = 373,185).

**Table 3:** Eligibility criteria for the CANVAS program (canagliflozin) and number of patients in the SIDIAP database that would have met criteria for enrolment.

Eligibility criteria in the CANVAS program	Potentially eligible patients from the SIDIAP database (*n* = 373,185)	
Inclusion criteria	In PP, *n* (%)	In SP, *n* (%)
Age:		
≥50 years in PP	349,896 (93.8)	—
≥30 years in SP	—	372,764 (99.9)
Primary prevention cohort		
≥50 years and ≥2 CVRF: (i) T2D duration ≥10 years (ii) Hypertension (iii) Current smoker (iv) Micro- or macroalbuminuria (v) HDLc − <39 mg/dl (1 mmol/l)	189,969 (50.9)	—
Secondary prevention cohort		
≥30 years and history of CV events (CHD, angina, MI, stroke, and PAD)	—	86,531 (23.2)
HbA1c: 7.0%-10.5%	83,537 (22.4)	34,320 (9.2)
Main exclusion criteria	In PP, *n* (%)	In SP, *n* (%)
eGFR < 30 ml/min/1.73m^2^	4731 (1.27)	2575 (0.69)
Total eligible in PP and SP, *n* (%)	**78,806 (21.1)**	**31,745 (8.5)**
Total eligible, *n* (%)	**110,551 (29.6)**	

CHD: coronary heart disease; CV: cardiovascular; CVRF: cardiovascular risk factors; eGFR: estimated glomerular filtration rate; MI: myocardial infarction; PP: primary cardiovascular prevention; SP: secondary cardiovascular prevention; PAD: peripheral artery disease; T2D: type 2 diabetes; HbA1c: glycated haemoglobin; HDLc: high-density lipoprotein cholesterol. All the percentages refer to the proportion from the total number of eligible subjects of the SIDIAP database (*n* = 373,185).

**Table 4:** Eligibility criteria for the DECLARE-TIMI 58 trial (dapagliflozin) and number of patients in the SIDIAP database that would have met criteria for enrolment.

Eligibility in the DELCLARE-TIMI 58 trial	Potentially eligible patients from the SIDIAP database (*n* = 373,185)	
Inclusion criteria	In PP, *n* (%)	In SP, *n* (%)
Age:		
(i) In PP: ≥55 years; ≥60 in women (ii) In SP: ≥40	175,092 (46.9); 140,941 (37.8) —	— 368,963 (98.9)
Primary prevention cohort		
≥55 years (≥60 in women) and ≥1 CVRF: (i) Dyslipidemia (ii) Hypertension (iii) Current smoker	289,126 (77.5)	—
Secondary prevention cohort		
≥40 years and history of CV events (CHD, angina, MI, stroke, and PAD)	—	86,468 (23.2)
HbA1c 6.5%-<12%	165,777 (44.4)	50,872 (13.6)
Main exclusion criteria	In PP	In SP
eGFR < 60 ml/min/1.73m^2^	53,851 (14.4)	21,145 (5.7)
Total eligible in PP and SP, *n* (%)	**111,926 (30)**	**29,727 (7.9)**
Total eligible, *n* (%)	**141,653 (38%)**	

CHD: coronary heart disease; CV: cardiovascular; CVRF: cardiovascular risk factors; eGFR: estimated glomerular filtration rate; MI: myocardial infarction; PAD: peripheral artery disease; PP: primary cardiovascular prevention; SP: secondary cardiovascular prevention; HbA1c: glycated haemoglobin. All the percentages refer to the proportion from the total number of eligible subjects of the SIDIAP database (*n* = 373,185).

**Table 5:** Summary of characteristics of patients enrolled in SGLT2i CVOTs and of patients from the SIDIAP database that would have met the corresponding criteria.

Characteristics	EMPA-REG OUTCOME	CANVAS	DECLARE-TIMI 58	General population (SIDIAP database)
Drug	Empagliflozin	Canagliflozin	Dapagliflozin	Empagliflozin/canagliflozin/dapagliflozin
Participants, *n*	7,020	10,142	17,160	373,185
Male (%)	71.5	64.2	62.6	54.9
Age (years), mean (SD)	63.1 (8.7)	63.3 (8.3)	63.9 (6.8)	70.1 (12.3)
Patients with established CVD, *n* (%)	7,020 (>99)	6,656 (66)	6,974 (41)	EMPA-REG OUTCOME criteria: 86,534 (23.2) CANVAS criteria: 86,531 (23.2) DECLARE-TIMI 58 criteria: 86,468 (23.2)
CVRFs, *n* (%)	—	3486 (34)	10,186 (59)	CANVAS criteria: 189,969 (50.9) DECLARE-TIMI 58 criteria: 289,126 (77.5)
HbA1c (%), mean (SD)	8.1 (0.8)	8.2 (0.9)	8.3 (1.2)	7.12 (1.32)
eGFR (ml/min/1.73 m^2^), *n* (%)				
≥60	5,199 (74.1)	8,114 (79.9)	15,959 (92.6)	241,958 (72)
<60-30	1,819 (25.9)	2,028 (20.1)	1,201 (7.4)	80,978 (24)
<30	0	0	0	13,262 (4)

CVD: cardiovascular disease; CVRF: cardiovascular risk factors; eGFR: estimated glomerular filtration rate; SD: standard deviation; CVOTs: cardiovascular outcome trials.

## Data Availability

The data used to support the findings of this study are available from the corresponding authors upon request.

## Supplementary Materials

Supplementary Materials Supplementary Table 1: eligibility criteria of the three studied CVOTs completed for SGLT2i in T2D patients. Supplementary Table 2: ICD-10 codes for cardiovascular diseases considered in the study.
