# Supplementary material for: How Many Patients with Type 2 Diabetes Meet the Inclusion Criteria of the Cardiovascular Outcome Trials with SGLT2 Inhibitors? Estimations from a Population Database in a Mediterranean Area
Source: J Diabetes Res. 2019 Nov 11;2019:2018374. doi: 10.1155/2019/2018374 (PMC6877986; doi:10.1155/2019/2018374)
Supplement: Supplementary Materials — Supplementary Table 1: eligibility criteria of the three studied CVOTs completed for SGLT2i in T2D patients. Supplementary Table 2: ICD-10 codes for cardiovascular diseases considered in the study. [file 2018374.f1.pdf]

Supplementary Table 1. Eligibility criteria of the three studied CVOTs completed for SGLT2i in T2D patients.

	**EMPA-REG OUTCOME (empagliflozin / placebo)**	**CANVAS (canagliflozin / placebo)**	**DECLARE-TIMI 58 (dapagliflozin / placebo)**
**Age**	≥18 years	• ≥30 years in SP<br>• ≥50 years in PP	• ≥40 years in SP<br>• ≥55 years (≥60 in women) in PP
**HbA1c**	• If no glucose-lowering agents: 7.0%-9.0%<br>• If glucose-lowering agents: 7.0%-10.0%	≥7.0% - ≤10.5%	6.5%-12.0%
**Main inclusion criteria**	Established CVD:<br>• Myocardial infarction<br>• CAD<br>• Unstable angina<br>• Stroke<br>• PAD	≥30 years with established CVD (SP):<br>• Ischaemic heart disease<br>• Myocardial infarction<br>• Unstable angina<br>• Significant carotid or PAD<br><br>≥50 years with high risk of CVD: ≥2 CV risk factors (PP):<br>• ≥10 years T2D duration<br>• Hypertension*<br>• Current smoker<br>• Micro or Macroalbuminuria<br>• HDL-c <39 mg/dl (<1 mmo/l)	≥40 years with established CVD (SP):<br>• Ischaemic heart disease<br>• Cerebrovascular disease<br>• PAD<br><br>High risk of CVD; ≥2 CV risk factors (PP):<br>• **Age AND** at least 1 of:<br>• Dyslipidemia**<br>• Hypertension†<br>• Current tobacco use
**Main exclusion criteria**	eGFR <30 ml/min/1.73 m^2	eGFR <30 ml/min/1.73 m^2	eGFR <60 ml/min/1.73 m^2
	BMI ≥45 kg/m^2		

CAD, coronary artery disease; CVD, cardiovascular disease; eGFR, estimated glomerular fraction; HbA1c, glycated haemoglobin; HDL-c, high-density lipoprotein-cholesterol; PAD, peripheral artery disease; PP, primary prevention; SP, secondary prevention; T2D, type 2 diabetes
*Systolic pressure >140 mmHg while on ≥1 hypertensive agents
**low-density lipoprotein-cholesterol (LDL-c) ≥130 mg/dl or use of lipid-lowering therapies
†Blood pressure >140/90 mmHg or on anti-hypertensive therapy

Supplementary Table 2. ICD-10 codes for cardiovascular diseases considered in the study.

Condition	ICD-10 codes
**Acute myocardial infarction (<28 days)**	I21
**Old myocardial infarction**	I25.2
**Angor pectoris**	I20.8
**Unstable angina**	I20.0
**Coronary artery disease**	I20-I25
**Stroke**	I61, I62.x (except: I62.9), I63, I64, I65.x, I67, I69, G45, and G46
**Peripheral artery disease**	I73, I73.8, I73.9, and I74
**Heart failure**	I50, I11.0, I13.0, and I13.2
**Procedures**	
Coronary revascularisation	Z95.5, Z95.8, Z95.9
Revascularisation of other vascular territories	I97.8, I97.9
